# Prognosis of alcohol-associated lactic acidosis in critically ill patients: an 8-year study

**DOI:** 10.1038/srep35368

**Published:** 2016-10-17

**Authors:** Chun-Chieh Yang, Khee-Siang Chan, Kuei-Ling Tseng, Shih-Feng Weng

**Affiliations:** 1 Department of Intensive Care Medicine, Chi Mei Medical Center, Tainan, Taiwan; 2 Department of Respiratory Therapy, Chi Mei Medical Center, Tainan, Taiwan; 3 Department of Healthcare Administration and Medical Informatics, Kaohsiung Medical University, Kaohsiung, Taiwan

## Abstract

Lactic acidosis is common in critical care; by contrast, a subtype called alcohol-associated lactic acidosis (AALA) is rarely encountered. The primary purpose of this study was to determine the prognosis of AALA in critically ill patients and the second aim was to determine whether the survival was associated to the peak blood lactate concentration. An 8-year retrospective analysis of adult patients admitted to the intensive care unit (ICU) with AALA between January 2007 and December 2014 was considered in a tertiary care hospital. In total, 23 patients were analyzed and the median peak blood lactate level was 15.9 mmol/L. Only 2 patients (8.7%) presented peak blood lactate levels <10 mmol/L. In this study, 21 patients survived from ICU and hospital, the mortality rate was 8.7%. The result indicted the survival of AALA was not associated with peak blood lactate concentration although survivors still had a better lactate clearance rate per hour than non-survivors. Moreover, AALA patients with coexisting sepsis presenting higher lactate clearance rate and shorter lactate clearance time than those of AALA patients with solely sepsis-related lactic acidosis.

Lactic acidosis is often encountered in intensive care unit (ICU) and can be associated with a variety of disease states, drugs and toxins including ethanol ingestion^1^. In view of biochemistry of lactate formation, lactic acidosis can be classified into two subtypes: Type A is associated with hypoperfusion or tissue hypoxia, related to imbalance of oxygen delivery and oxygen consumption, while, Type B includes conditions affecting the production and elimination of lactate, unrelated to oxygen debt^2^,^3^. The potential seriousness of lactic acidosis is its strong association with increased mortality^4^,^5^,^6^,^7^,^8^,^9^. A blood lactate concentration in excess of 5 mmol/L on ICU admission is associated with a 3- and 30-day mortality rate of 59% and 83%, respectively^10^.

In fact, lactic acid is produced and metabolized in all cells of the body except red blood cells, which have no mitochondria^11^. Review of the formation of lactic acid shows that glucose is broken down to pyruvate through glycolysis, and pyruvate is then converted to lactate as the end product of anaerobic metabolism. On the other hand, lactates can only be metabolized via their conversion back to pyruvate^12^. In addition, ethanol is mainly metabolized in the liver by alcohol dehydrogenase. After being oxidized to acetaldehyde, acetaldehyde is further oxidized to acetic acid by aldehyde dehydrogenase. Both enzymatic steps result in the generation of reduced form of nicotinamide adenine dinucleotide dehydrogenase (NADH). The increase of the NADH/NAD ratio favors the metabolism of pyruvate to lactate; furthermore, the elimination of lactate depends mainly on the conversion of pyruvate to acetyl CoA, gluconeogenesis, and its uptake by the liver and kidneys. This conversion of pyruvate to acetyl CoA requires the enzyme called pyruvate dehydrogenase and the coenzyme called thiamine. Therefore, chronic alcoholism and associated malnutrition may also decrease lactate utilization due to inadequate intake of the water-soluble vitamins such as thiamine and biotin. Theoretically both the metabolism of ethanol and the deficiency of thiamine tend to drive the formation of lactate and subsequently lead to the result of severe lactic acidosis in patients with acute or chronic abuse of alcohol. The primary purpose of this study was to determine the prognosis of alcohol-associated lactic acidosis (AALA) in critically ill adult patients, and the second aim was to verify whether the survival was associated to the peak blood lactate concentration.

## Methods

### Study sample

This was a retrospective observational study in the ICUs (96 beds) of a tertiary care medical center. Patients who were admitted to the ICU with the diagnosis of AALA between January 2007 and December 2014 were recruited into this study. AALA refers to patients with alcohol abuse and a blood lactate level >5 mmol/L. Sometimes the diagnosis was considered by the clinicians in charge despite the level of serum alcohol. Patients who had an AALA history before January 2007 were excluded. This study was approved by the Institutional Review Board of Chi Mei Medical Center (IRB-10407-002) and waived the need for informed consent. The methods were carried out in accordance with the approved guidelines.

All clinical and laboratory data at ICU admission and ICU stay were recorded, including: (1) Clinical characteristics: age, gender, Acute Physiology and Chronic Health Evaluation II (APACHE II) score, hypoglycemic event, seizure episode, coexisting diseases such as gastrointestinal (GI) bleeding, sepsis, liver disease (liver cirrhosis, hepatitis), acute kidney injury, chronic kidney disease and the status of ICU and hospital discharge; (2) Laboratory characteristics: peak blood lactate level, dynamic blood lactate level when blood lactate concentration was <5 mmol/L, time interval by hours between the peak and dynamic blood lactate level, nadir arterial PH and bicarbonate level, presence of ketone bodies in either urine or blood specimens, serum ethanol level, serum methanol level, presence of high mean corpuscular volume (MCV) of red blood cell, and presence of hyperammonemia; (3) Treatment modalities: during the treatment period of lactic acidosis from blood lactate level >5 mmol/L to <5 mmol/L, the clinical need of vasoactive support, ventilator support, and renal replacement therapy (hemodialysis or continuous veno-venous hemofiltration) was also recorded. All the definitions of variables were listed in Table 1.

### Statistical analysis

The clinical characteristics of patients with AALA were both expressed as mean and standard deviation and median with interquartile range (IQR) for continuous variables and percentage frequency for categorical variables. For continuous variables, Wilcoxon rank-sum tests were used to compare the difference between survivor and non-survivor sub-groups due to the small sample sizes. Pearson’s chi-square tests were applied to compare the category data between groups. If 20% of the cells had expected frequencies of less than 5, Fisher’s exact tests was used. The significant level was set at 0.05 for the two sided test. SAS for Windows 9.4 (SAS Institute, Inc, Cary, NC, USA) was performed for all analyses.

## Results

During this study period, twenty-three patients were admitted to the ICU with AALA. The clinical and laboratory characteristics of these patients were shown in Table 2 and most of patients were male (n = 22, 22/23 = 95.7%). The median peak blood lactate level was 15.9 mmol/L (IQR = 8.2 mmol/L). There were only 2 patients (8.7%) presenting peak blood lactate level <10 mmol/L; other 21 patients (91.3%) were with blood lactate concentration >10 mmol/L, including 8 patients >10 but ≦15 mmol/L as well as 5 patients >15 but ≦20 mmol/L. Other 8 patients had a peak blood lactate level of >20 mmol/L.

Blood or urine ketone body test was performed in 20 patients and 15 (75.0%) of them showed positive results. Serum alcohol level was checked in 14 patients and positive results were found in 10 patients. No patients had seizure episode or methanol intoxication. Most patients presented hyperammonemia (73.9%) and high MCV (60.9%). In addition, 16 patients (69.6%) presented coexisting acute kidney injury while 13 patients (56.5%) were with liver disease and 11 patients (47.8%) had sepsis.

The comparison between the survivors and non-survivors was demonstrated in Table 3. There were 21 patients survived from the ICU and hospital. The mortality rate in our study was 8.7%. In addition, the survival of AALA was not associated with the peak blood lactate concentration.

## Discussion

This study presented 23 patients admitted to the ICU with AALA in an 8-year period, and 21 patients survived from the ICU and hospital. Between survivors and non-survivors, the peak blood lactate concentration (Median = 17.8 vs 14.6, P-value = 0.711) and APACH II (Median = 14.0 vs 20.5, P-value = 0.176) score among these two sub-groups were not associated with the survival of AALA.

This study demonstrated an unusual finding that the survivors had lower nadir arterial blood pH compared to the non-survivors because of the small number of the non-survivors and metabolic acidosis presented in these two patient sub-groups was respiratory overcompensated by the use of mechanical ventilation. In addition, in comparison with non-survivors, survivors had lower APACHE II scores but higher peak blood lactate concentrations, though they did not reach statistical significance. Our results were inconsistent with the previous literature, which emphasized the potential seriousness of lactic acidosis and proved a strong association with increased mortality^4^,^5^,^6^,^7^,^8^,^9^. However, several case reports also showed a favorable outcome of patients with AALA who had similar high blood lactate level^13^,^14^,^15^,^16^. This finding might reflect the entity of AALA (type B lactic acidosis), which actually was not related to the balance of oxygen delivery and oxygen consumption. In our study, 10 patients with peak blood lactate level between 5 and 15 mmol/L did not have the need of vasoactive support during the treatment period of AALA. Moreover among 13 patients who had blood lactate level >15 mmol/L, only 3 of them needed vasoactive support.

Considering the relationship between the arterial blood PH, bicarbonate concentration and blood lactate level among these 23 AALA patients, we found that some patients had a blood lactate concentration of >5 mmol/L and the simultaneous arterial blood PH was >7.35. In fact, for those 4 patients whose blood lactate level >10 but ≦15 mmol/L, their coexisting PH levels were 7.43, 7.47, 7.36, and 7.53, respectively. Arterial blood PH levels of 7.43, 7.47 and 7.53 were the combination of metabolic alkalosis and respiratory alkalosis while PH level of 7.36 was caused by mixed type of metabolic acidosis and respiratory alkalosis. One patient who was presenting blood lactate level >15 mmol/L had coexisting PH of 7.37, which resulted from metabolic acidosis and respiratory alkalosis. Further discussions regarding the cause of respiratory alkalosis are warranted. Furthermore, the effect of mechanical ventilation was seen in patients due to their arterial blood PH levels of 7.35 and 7.53 instead of 7.37, 7.43 and 7.47. The presence of mixed type acid-base disturbances is common in critical care. Therefore, it is not feasible to predict the severity of lactic acidosis just by using the arterial blood PH and bicarbonate level.

The reason that alcohol should be considered is its important role in lactic acidosis diagnosed by the clinicians, although the reasons that the coexisting medical illnesses such as GI bleeding, sepsis, liver disease (liver cirrhosis, hepatitis), acute kidney injury and chronic kidney disease might affect the severity and clinical manifestations of lactic acidosis were not yet completely explained. Consider the contribution of AALA, 10 patients with peak blood lactate level between 5 and 15 mmol/L did not have the need of vasoactive support. Even in 13 patients with lactate level >15 mmol/L, only 3 patients needed vasoactive support. Of these AALA patients, no patients needed ventilator support when peak lactate level was ≦10 mmol/L, although 2 out of those 21 patients with blood lactate concentration >10 mmol/L needed ventilator support. Similarly, no patients needed renal replacement therapy when blood lactate concentration was ≦20 mmol/L. However, 1 patient underwent renal replacement therapy while the peak blood lactate concentration was 35.8 mmol/L.

According to previous study results regarding the discussion between the lactate clearance and patient prognosis^17^,^18^,^19^,^20^,^21^,^22^,^23^,^24^,^25^,^26^, our study reflected similar findings, i.e. in comparison with non-survivors, a better lactate clearance was found in survivors. The average lactate clearance rate per hour was 10.1% ± 6.6% in survivors versus 3.6% ± 1.1% in non-survivors. In addition, this study revealed an important finding that higher lactate clearance rate and shorter lactate clearance time were found in patients with AALA and coexisting sepsis than those with solely sepsis-related lactic acidosis. In the report showed by Marty *et al*.^26^ the 24 hours lactate clearance in patients with severe sepsis or septic shock was 42% ± 33% in survivors and −17% ± 76% in non-survivors, where the lactate clearance rate was 85.1% ± 8.9% (66.2% to 93.3%) in patients with AALA along with sepsis in our study, and the average duration was 14 hours and 42 minutes.

There was a question of the serum alcohol level in patients presenting AALA. In this study, positive results were detected in 10 patients; however, the serum alcohol level was not an absolute indication for the diagnosis of AALA because either acute or chronic alcohol abuse, only whether alcohol intake was continued until the time of presentation mattered. Besides, all 23 AALA patients received thiamine therapy. Furthermore, our study showed that 14 patients had high MCV, which could be considered macrocytosis, and excessive alcohol intake itself or the combination with coexisting acute GI bleeding and liver disease could be the common causes and thus played important roles. In addition, among 17 patients who presented hyperammonemia, 11 of them were with liver diseases, which probably were related to excessive alcohol consumption as well.

There were several limitations in our study. First, this was a single center, retrospective observational study. Second, the patient’s number was small. Third, AALA was defined as patients with alcohol abuse combining a blood lactate level >5 mmol/L, and the diagnosis had been considered by the clinicians in charge despite the serum alcohol level, even though the coexisting medical illnesses such as GI bleeding, sepsis, liver disease (liver cirrhosis, hepatitis), acute kidney injury and chronic kidney disease may also were contributed to lactic acidosis. Although it is not easy to calculate the real contribution of alcohol to the severity of lactic acidosis on AALA, the role of alcohol could not be underemphasized after consideration of 2 patients (8.7%) presenting peak blood lactate level <10 mmol/L; other 21 patients (91.3%) were with blood lactate concentration >10 mmol/L, including 8 patients >10 but ≦15 mmol/L as well as 5 patients >15 but ≦20 mmol/L and other 8 patients had a peak blood lactate level >20 mmol/L. Among these 23 patients, only 3 patients needed vasoactive support, ventilator support in 2 patients, and renal replacement therapy in 1 patient from blood lactate level >5 mmol/L to <5 mmol/L.

To our knowledge, this study is the largest case series ever reported on critically ill patients with AALA, and the results show that the prognosis of AALA is better than the predicted outcome according to the severity of lactic acidosis. There were several case reports also showed a favorable outcome of patients with AALA who had similar high blood lactate level^13^,^14^,^15^,^16^. Even though the peak blood lactate concentration is not associated with the survival of AALA; however, survivors had better lactate clearance rate per hour than non-survivors and AALA patients with coexisting sepsis presenting higher lactate clearance rate and shorter lactate clearance time than those had solely sepsis-related lactic acidosis.

## Additional Information

**How to cite this article**: Yang, C.-C. *et al*. Prognosis of alcohol-associated lactic acidosis in critically ill patients: an 8-year study. *Sci. Rep.* **6**, 35368; doi: 10.1038/srep35368 (2016).

## Figures and Tables

**Table 1:** Definition of variables.

Variable	Definition
Alcohol abuse	Uncontrolled alcohol drinking despite adverse consequences such as hepatic toxicity.
Hypoglycemic event	Plasma glucose <70 mg/dL with or without symptoms^27^.
Acute kidney injury	At least 1.5 times increase in baseline serum creatinine.
Chronic kidney disease	Baseline serum creatinine ≧1.5 mg/dL for men and ≧1.4 mg/dL for women.
Liver disease	The presence of liver cirrhosis or hepatitis. Cirrhosis is diagnosed according to clinical manifestations, laboratory results and image studies; and hepatitis is diagnosed using the evidence regarding abnormal hepatic biochemical and functional tests.
Sepsis	The presence of infection together with systemic manifestations of infection.
High MCV	MCV >100 fL (normal range of MCV: 80–100 fL).
Hyperammonemia	Serum ammonia level >32 μmol/L (normal range of ammonia level: 11–32 μmol/L).
Lactate clearance rate per hour	100% **× **[(peak blood lactate level - dynamic blood lactate level when blood lactate concentration <5 mmol/L)/(peak blood lactate level)]/(time interval by hours spent between the peak and dynamic blood lactate level).

**Table 2:** Clinical and laboratory characteristics.

Characteristics	Values (N = 23)		
	n (%)	Median/IRQ	mean ± SD
Age (years)		51.0/10.0	50.0 ± 10.9
APACHE II score		14.0/9.0	15.5 ± 6.7
Peak lactate level (mmol/L)		15.9/8.2	17.9 ± 7.0
Nadir arterial pH		7.25/0.34	7.18 ± 0.21
Nadir arterial bicarbonate level (mmol/L)		8.7/11.3	9.5 ± 5.8
Sex, male, (%)	22 (95.7%)		
Positive blood or urine ketone body, n (%) (N = 20)	15 (75.0%*)		
Positive serum alcohol level, n (%) (N = 14)	10 (71.4%$)		
Hypoglycemia, n (%)	2 (8.7%)		
Hyperammonemia, n (%)	17 (73.9%)		
High MCV, n (%)	14 (60.9%)		
Gastrointestinal bleeding, n (%)	5 (21.7%)		
Sepsis, n (%)	11 (47.8%)		
Acute kidney injury, n (%)	16 (69.6%)		
Chronic kidney disease, n (%)	2 (8.7%)		
Liver cirrhosis, n (%)	9 (39.1%)		
Hepatitis, n (%)	4 (17.4%)		
Need of vasoactive support, n (%)	3 (13.0%)		
Need of ventilator support, n (%)	2 (8.7%)		
Need of renal replacement therapy, n (%)	1 (4.3%)		

^*^Based on 20 cases.

^$^Based on 14 cases.

**Table 3:** The comparison between survivors and non-survivors.

Characteristics	Survivors Median/IRQ (min~max)	Non- survivors Median^$^ (min~max)	p
No. of patients	21	2	
Age (years)	51.0/10.5 (32–78)	45.5 (40–51)	0.561
Sex, male, n (%)	20 (95.2%)	2 (100%)	1.000
APACHE II score	14.0/10 (7–33)	20.5 (17–24)	0.176
Peak lactate level (mmol/L)	17.8/8.8 (9.7–35.8)	14.6 (13.3–15.9)	0.711
Lactate clearance rate per hour	7.4/6.9 (3.0–25.3)	3.5 (2.8–4.3)	0.047*
Nadir arterial pH	7.2/0.3 (6.8–7.5)	7.45 (7.4–7.5)	0.042*
Nadir arterial bicarbonate level (mmol/L)	8.2/8.5 (2.3–21.1)	15.5 (13.3–17.6)	0.158
Detectable blood or urine ketone body, n (%)	15 (78.9%)	0 (0%)	0.250
Detectable serum alcohol level, n (%)	10 (71.4%)	—	—
Hypoglycemia, n (%)	2 (9.5%)	0 (0%)	1.000
Hyperammonemia, n (%)	15 (71.4%)	2 (100%)	1.000
High MCV, n (%)	13 (61.9%)	1 (50%)	1.000
Gastrointestinal bleeding, n (%)	4 (19%)	1 (50%)	0.395
Sepsis, n (%)	9 (42.9%)	2 (100%)	0.217
Acute kidney injury, n (%)	15 (71.4%)	1 (50%)	0.526
Chronic kidney disease, n (%)	2 (9.5%)	0 (0%)	1.000
Liver cirrhosis, n (%)	7 (33.3%)	2 (100%)	0.142
Hepatitis, n (%)	4 (19%)	0 (0%)	1.000
Vasoactive support, n (%)	3 (14.3%)	0 (0%)	1.000
Mechanical ventilation support, n (%)	0 (0%)	2 (100%)	<0.05*
Renal replacement therapy, n (%)	1 (4.8%)	0 (100%)	1.000

Wilcoxon rank-sum tests were used for continuous variables and Fisher’s exact tests was used for categorical variables.

*A statistically significant between-group difference (P < 0.05).

^$^Due to only 2 cases, no IQR performed.
